# Supplementary material for: Depuration of Asian Green Mussels Using Chitooligosaccharide-Epigallocatechin Gallate Conjugate: Shelf-Life Extension, Microbial Diversity, and Quality Changes during Refrigerated Storage
Source: Foods. 2024 Sep 28;13(19):3104. doi: 10.3390/foods13193104 (PMC11476230; doi:10.3390/foods13193104)
Supplement: Supplementary file 1 [file foods-13-03104-s001.zip › foods-3214393-supplementary.pdf]

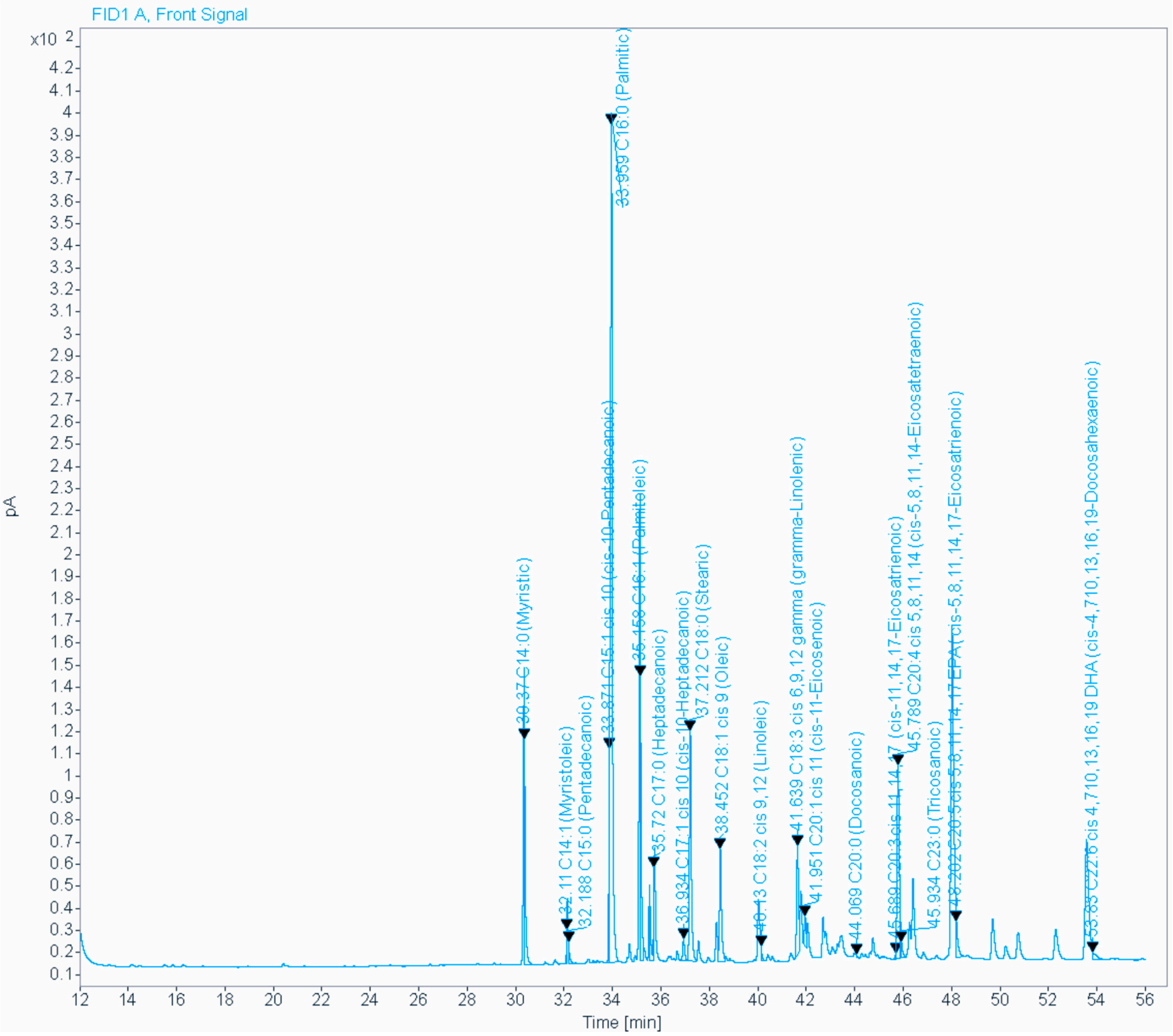

Figure S1-A. Fatty acid profile of Asian green mussels (AGM) treated without COS-EGCG conjugate (CEC) at day 0 during storage at 4 °C.

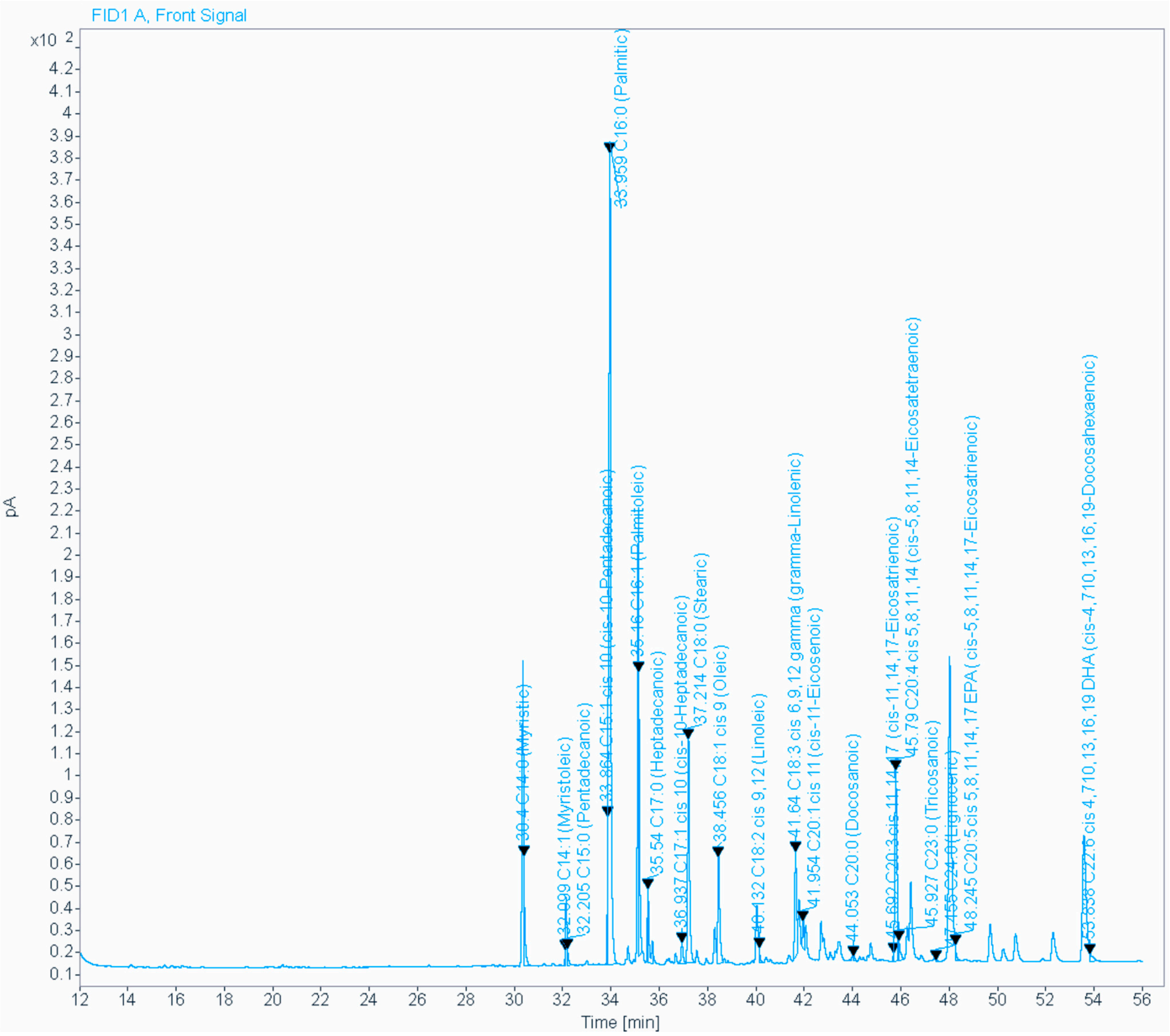

Figure S1-B. Fatty acid profile of Asian green mussels (AGM) treated with 2% COS-EGCG conjugate (CEC) at day 0 during storage at 4 °C.

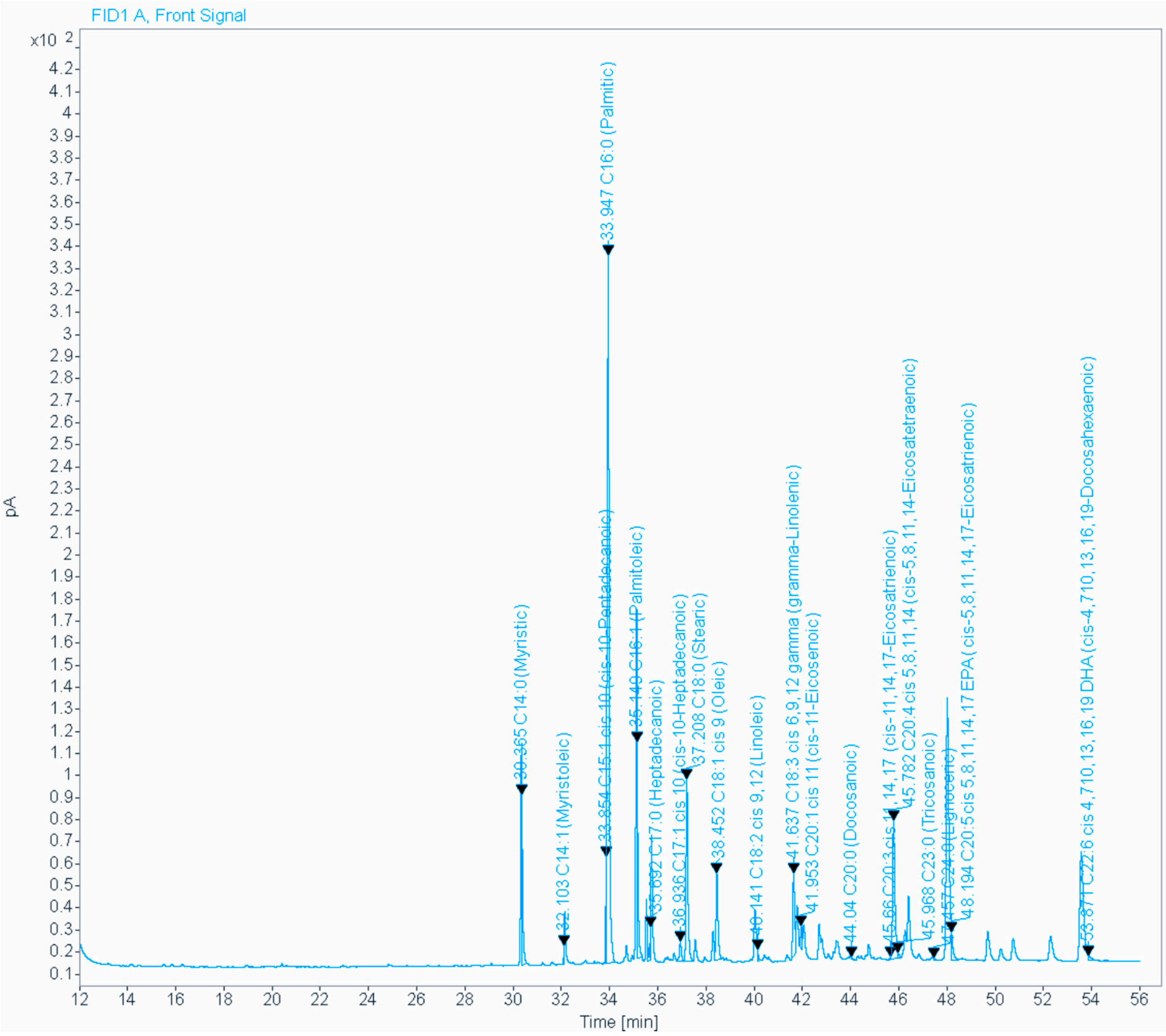

Figure S1-C. Fatty acid profile of Asian green mussels (AGM) treated with 2% COS-EGCG conjugate (CEC) at day 4 during storage at 4 °C.
